# On compressible and piezo-viscous flow in thin porous media

**DOI:** 10.1098/rspa.2017.0601

**Published:** 2018-01-03

**Authors:** F. Pérez-Ràfols, P. Wall, A. Almqvist

**Affiliations:** 1 Division of Machine Elements, Luleå University of Technology, 97187 Luleå, Sweden; 2 Department of Mathematics, Luleå University of Technology, 97187 Luleå, Sweden

**Keywords:** Reynolds equation, thin film flow, compressible fluid, piezo-viscous fluid

## Abstract

In this paper, we study flow through thin porous media as in, e.g. seals or fractures. It is often useful to know the permeability of such systems. In the context of incompressible and iso-viscous fluids, the permeability is the constant of proportionality relating the total flow through the media to the pressure drop. In this work, we show that it is also relevant to define a constant permeability when compressible and/or piezo-viscous fluids are considered. More precisely, we show that the corresponding nonlinear equation describing the flow of any compressible and piezo-viscous fluid can be transformed into a single linear equation. Indeed, this linear equation is the same as the one describing the flow of an incompressible and iso-viscous fluid. By this transformation, the total flow can be expressed as the product of the permeability and a nonlinear function of pressure, which represents a generalized pressure drop.

## 1. Introduction

**Figure 1.**
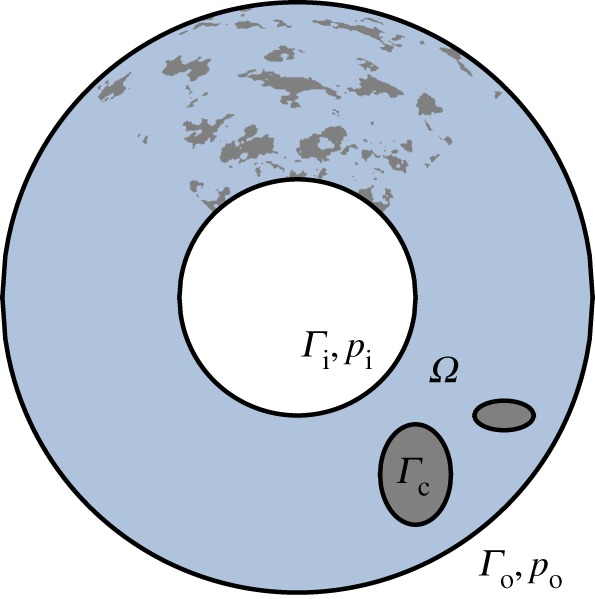
Schematic of the fluid flow domain of a seal, *Ω*, and its boundary, *Γ*. It includes an inlet *Γ*_*i*_, at a pressure *p*_*i*_, an outlet *Γ*_*o*_, at a pressure *p*_*o*_, and the boundary of the contact patches, *Γ*_*c*_. In the upper part a more realistic contact pattern is depicted. (Online version in colour.)

Leakage of seals has interested several researchers, both experimentally and numerically [1–6]. The function of a seal is to prevent the fluid flow through the rough aperture between the sealing surfaces. More generally, the leakage problem can be seen as a special problem regarding flow through thin porous media ([7, ch. 4] or [8]). When studying leakage numerically or analytically, most studies have focused on the case where the fluid’s density and viscosity are constant. In this case, the problem becomes linear and several simplifications can be done to study the problem. Figure 1 schematically depicts the flow domain *Ω* for a typical seal. The fluid flows through this domain from the inlet boundary, *Γ*_*i*_ to the outlet boundary, *Γ*_*o*_, driven by a pressure difference Δ*p*=*p*_*i*_−*p*_*o*_, where *p*_*i*_ and *p*_*o*_ are the pressures at the inlet and outlet boundaries, respectively. This pressure difference gives rise to the total mass flow *Q* (across the outlet boundary *Γ*_*o*_). For incompressible and iso-viscous fluids, this total mass flow can be modelled with Darcy’s Law:

1.1 $$Q=K\frac{\rho \Delta p}{\eta },$$

where *ρ* and *η* are the density and dynamic viscosity of the fluid. The proportionality constant *K* is referred to as the permeability of the seal and only depends on the gap between the contacting rough surfaces. Note that, once the permeability is known, it is possible to compute the total mass flow, *Q*, for all possible values of the pressure drop, density and viscosity.

As pressure increases, however, one can no longer consider density nor viscosity as constant. Furthermore, it has been shown in the field of lubrication that a complex rheology must be used in order to obtain realistic results (e.g. [4,9]). If density and viscosity are allowed to depend on pressure, the flow problem becomes nonlinear. In this work, we show that the nonlinear problem can be transformed into a linear one. The same technique has recently been used in [9,10], considering cavitation in hydrodynamic lubrication. In our case, this transformation allows us to formulate a generalized Darcy’s Law, in which the permeability is the same as in (1.1) and depends only on the topography of the rough aperture between the surfaces and the global geometry of the seal. The nonlinearity now appears as a generalized pressure drop WHICH includes information regarding the dependency of viscosity and density with pressure. This formulation has the advantage that the leakage, *Q*, for all types of Newtonian, compressible and piezo-viscous fluids, may be computed via the solution of a single linear problem (3.1).

## 2. Problem definition

Taking as a reference the example presented in figure 1, let us now formulate the problem in a general manner.

In this work, we assume that the thin film approximation holds. Furthermore, we assume the flow to be isothermal and that density and viscosity only depend on pressure. Under these assumptions, the pressure in the fluid, *p*, can be modelled by the following nonlinear problem:

2.1*a* $$\begin{array}{r} \nabla \cdot \left(\frac{h^{3}\rho (p)}{12\eta (p)}\nabla p\right)=0\;\text{in}\;\Omega , \end{array}$$

2.1*b* $$\begin{array}{r} p=p_{i}\;\text{on}\;\Gamma_{i}, \end{array}$$

2.1*c* $$\begin{array}{r} p=p_{o}\;\text{on}\;\Gamma_{o} \end{array}$$

2.1*d* $$\begin{array}{r} \;\text{and}\;h^{3}\nabla p\cdot n_{\Gamma }=0\;\text{on}\;\Gamma_{c}, \end{array}$$

where *h* is the gap between the surfaces, *ρ*(*p*) is the density of the fluid, *η*(*p*) is its viscosity and *n*_*Γ*_ is the unit vector normal to the boundary. In (2.1), *Γ*_*c*_ is the boundary of the contact patches. It is important to clarify that the geometry depicted in figure 1 only serves as an example. What is critical for the result presented is that the pressure *p*_*i*_ at the boundary *Γ*_*i*_ is constant and similarly the pressure *p*_*o*_ at the boundary *Γ*_*o*_. At the rest of the boundaries, *Γ*_*c*_, zero flux should be specified. Why these restrictions on the boundary conditions are critical will be made apparent in §4.

In some papers, other types of flow domains are studied. For example, in [2] a rectangular domain with periodic boundary conditions is considered. The analysis presented here can be applied also to this case.

## 3. The linear problem and the definition of permeability

The permeability in (1.1) can be defined by solving a linear problem, in which we assume *ρ*=1, *η*=1, *p*_*i*_=1 and *p*_*o*_=0. Note that the latter two can be thought of as expressing the pressure in a non-dimensional form. Thus, the linear problem can be formulated as:

3.1*a* $$\begin{array}{r} \nabla \cdot (h^{3}\nabla p)=0\;\text{in}\;\Omega , \end{array}$$

3.1*b* $$\begin{array}{r} p=1\;\text{on}\;\Gamma_{i}, \end{array}$$

3.1*c* $$\begin{array}{r} p=0\;\text{on}\;\Gamma_{o} \end{array}$$

3.1*d* $$\begin{array}{r} \;\text{and}\;h^{3}\nabla p\cdot n_{\Gamma_{c}}=0\;\text{on}\;\Gamma_{c}. \end{array}$$

Once (3.1) is solved, then the permeability can be computed as

3.2 $$K=\int_{\Gamma_{o}}\frac{h^{3}}{12}\nabla p\cdot n_{\Gamma_{o}}\;\text{d}s,$$

where *s* parametrizes *Γ*_*o*_. This reference problem can be in itself a hard one to solve. Indeed, many efforts have been directed in the literature to solve it more efficiently. The interested reader is referred to, e.g. [11,12] for a two-scale approach based on homogenization, [6] for a stochastic two-scale approach, [1] for an approximation based on the concept of critical constriction and [13] for an approach based on Bruggeman’s effective-medium theory. In this work, we will assume that a solution of this problem is known, either directly or by using some of the previously mentioned methods, and that the permeability is known. Based on that, we will investigate what we can say about the solution of (2.1).

## 4. Transformation of the nonlinear problem

Let us write the viscosity and pressure appearing in the nonlinear problem (2.1) as *η*=*η*_*o*_*f*_*η*_(*p*) and *ρ*=*ρ*_*o*_*f*_*ρ*_(*p*), respectively. The constants *η*_*o*_ and *ρ*_*o*_ are reference values, taken at the outlet pressure. In this case, the mass flow (keeping the thin film approximation in mind) can be expressed as

4.1 $$q=h^{3}\frac{\rho_{o}f_{\rho }(p)}{12\eta_{o}f_{\eta }(p)}\nabla p=h^{3}\frac{\rho_{o}}{12\eta_{o}}f(p)\nabla p.$$

Whenever the function *f*(*p*) is not a constant, the problem (2.1) is nonlinear. However, by a change of variables, it can be transformed into a linear problem. Indeed, define a function *F*(*p*) such that *dF*(*p*)/*dp*=*f*(*p*). Using this function, we introduce a new variable *u* in the following way:

4.2 $$u=c_{1}F(p)+c_{2}.$$

The gradient of this variable is now

4.3 $$\nabla u=c_{1}\frac{dF(p)}{dp}\nabla p=c_{1}f(p)\nabla p,$$

where *c*_1_ and *c*_2_ are constants. This allows us to rewrite the mass flow in (4.1) as

4.4 $$q=h^{3}\frac{\rho_{o}}{12\eta_{o}c_{1}}\nabla u.$$

We point out here that in order to perform the change of variables from *p* to *u*, we must require that the inverse function, *F*^−1^, exists. This is, however, always the case, as both density and viscosity are always positive and thus *dF*/*dp*=*f*(*p*)=*f*_*ρ*_(*p*)/*f*_*η*_(*p*)>0. Moreover, the constants *c*_1_ and *c*_2_ are chosen to obtain the same boundary conditions as in the linear problem (3.1). This leads to *c*_1_=1/(*F*(*p*_*i*_)−*F*(*p*_*o*_)) and *c*_2_=−*F*(*p*_*o*_)/(*F*(*p*_*i*_)−*F*(*p*_*o*_)). Hence,

4.5 $$u=\frac{F(p)-F(p_{o})}{F(p_{i})-F(p_{o})},$$

and, accordingly,

4.6 $$p=F^{-1}((F(p_{i})-F(p_{o}))u+F(p_{o})).$$

From (4.4), it follows that the zero flux condition in (3.1d) is equivalently expressed in terms of *p* and *u*. We note also that it is apparent from (4.5) that periodicity in *p* implies periodicity in *u* and vice versa. We, therefore, have that *u* solves the following linear problem:

4.7a $$\begin{array}{r} \nabla \cdot (h^{3}\nabla u)=0\;\text{in}\;\Omega , \end{array}$$

4.7b $$\begin{array}{r} u=1\;\text{on}\;\Gamma_{i}, \end{array}$$

4.7c $$\begin{array}{r} u=0\;\text{on}\;\Gamma_{o} \end{array}$$

4.7d $$\begin{array}{rl} \;\text{and}\;h^{3}\nabla u\cdot n_{\Gamma_{c}} & =0\;\text{on}\;\Gamma_{c}. \end{array}$$

Note that this is exactly the same linear problem as in (3.1), which described pressure for fluids with constant viscosity and density. Thus, once the solution of the linear problem (4.7) has been found, it can be used to compute the total mass flow (*Q*) for any type of fluid by means of the transformation (4.5) and (4.6). More precisely,

4.8 $$Q=\int_{\Gamma_{o}}q\cdot n_{\Gamma_{o}}\;\text{d}s=K\frac{\rho_{o}}{\eta_{o}}\Delta F,$$

where *K* is defined as in (3.2). Note that the only difference between this equation and (1.1) is that the pressure drop (Δ*p*) is replaced by the nonlinear expression

4.9 $$\Delta F=F(p_{i})-F(p_{o}),$$

which can be thought of as a generalized pressure drop. For constant density and viscosity, *f*(*p*)=1 and thus Δ*F*=Δ*p* and (4.8) becomes equal to (1.1).

## 5. Solution procedure

Summing up, we have derived the following solution procedure to compute the total mass flow (*Q*) and the associated pressure distribution; one should perform the following steps:

(i) Solve the linear problem (4.7) for *u* and obtain the permeability (*K*).
(ii) Compute *F*(*p*), analytically if possible, numerically otherwise.
(iii) Compute the pressure distribution using (4.6).
(iv) Compute the total mass flow (*Q*) using (4.8).

In table 1, we have listed some frequently used pressure–density and pressure–viscosity relations for which an analytical form of *F*(*p*) can be obtained. Note that, even in cases when it is not possible to obtain a closed-form expression for *F*(*p*), one can still numerically integrate *f*(*p*) to obtain *F*(*p*). Note also that, as *η*/*ρ* is the kinematic viscosity of the fluid, a constitutive equation relating it to the pressure can also be used. The procedure presented in this work can therefore be applied to any Newtonian compressible and piezo-viscous fluid.

**Table 1.** Overview of results for different fluid models.

	*f*(*p*)	*F*(*p*)	Δ*F*	*p*(*u*)
ideal gas	$\frac{p}{p_{o}}$	p2AA2poAAA	$\frac{p_{i}^{2}-p_{o}^{2}}{2p_{o}}$	$\sqrt{(p_{i}^{2}-p_{o}^{2})u+p_{o}^{2}}$
constant bulk modulus	*e*^*β*(*p*−*p*_*o*_)^	$\frac{1}{\beta }\;e^{\beta (p-p_{o})}$	eβ(pi−po)AA−1β	$\frac{1}{\beta }\log[(e^{\beta (p-p_{o})}-1)u+1]+p_{o}$
Barus’ Law	*e*^*α*(*p*−*p*_*o*_)^	$\frac{1}{\alpha }\;e^{\alpha (p-p_{o})}$	eα(pi−po)AA−1αAA	$\frac{1}{\alpha }\log[(e^{\alpha (p-p_{o})}-1)u+1]+p_{o}$
power law	${\left(\frac{p}{p_{o}}\right)}^{n}$	pn+1AA(n+1)pon	$\frac{p_{i}^{n+1}-p_{o}^{n+1}}{(n+1)p_{o}^{n}}$	${\left((p_{i}^{n+1}-p_{o}^{n+1})u+p_{o}^{n+1}\right)}^{1/(n+1)}$
Dowson and Higgingson’s	κ1+κ2(p−po)κ1+(p−po)AA	$\kappa_{2}(\kappa_{1}-(p-p_{o}))+\kappa_{1}(1-\kappa_{2}\log(\kappa_{1}+(p-p_{o})))$	$-\kappa_{2}(p_{i}-p_{o})+\kappa_{1}(1-\kappa_{2})\log(1+\frac{p_{i}-p_{o}}{\kappa_{1}})$	—

## 6. Application example: flow through saddle points

Even though it is not the main focus of this work, we will consider the result in [14] to demonstrate the applicability of the generalized expression for the mass flow, presented in (4.8). Dapp & Müser [14] study how the total flow, of an incompressible and iso-viscous fluid, through a sinusoidal gap evolves as the load closing it increases. They found out that a saddle point forms over which the entire pressure drop occurs. Although in [14] adhesion was also considered, here we focus only on the basic, adhesion-free case. In that work, it was concluded that, close to the percolation threshold, permeability follows a power law with the applied load *L*, i.e.

6.1 $$K\propto {\left(L-L_{c}\right)}^{\beta },$$

where *L*_*c*_ is the applied load at the percolation threshold and *β* is an empirical exponent which they found to be 3.45. This power law was shown to be valid also for gaps formed when a self-affine fractal surface contacts a flat one, near the percolation threshold [15]. Let us consider the situation that would occur if the incompressible and iso-viscous fluid is replaced by a compressible and/or piezo-viscous one. We realize that this would result in a quite complex flow situation, with the localized pressure drop implying substantial changes in both the density and the viscosity of the fluid around the saddle point. However, having either an analytical, semi-empirical or fully empirical description of *F*(*p*), as presented in §5, we can easily use (4.8) to compute the total flow in this situation, i.e.

6.2 $$Q=K\frac{\rho_{o}}{\eta_{o}}\Delta F\propto {\left(L-L_{c}\right)}^{\beta }\Delta F.$$

Indeed, we have shown how the present result can be used to generalize the results presented in [14,15] to be applicable to the flow of compressible and/or piezo-viscous fluids. In particular, we have shown that, in this case, the total flow also follows a power law with the applied load.

## 7. Concluding remarks

In this work, we present a generalized version of Darcy’s Law. In particular, we show that the leakage of a wide range of fluids can be expressed by a Darcy-type law where the pressure drop is replaced by a nonlinear generalized pressure drop. The mass flow for these fluids can be obtained from the solution of a single linear problem (i.e. the original Reynolds problem, assuming no pressure dependence of density or viscosity) and the knowledge of the integral of *ρ*(*p*)/*η*(*p*). The results presented herein can be applied to extend previous results. Examples of previous work addressing incompressible and iso-viscous fluid flow through thin gaps, which could be generalized by means of the present findings are presented in [1–6,14,15]. In particular, we have shown how the present study can be used to generalize the results in [15].

## Data accessibility

This article has no additional data.
